# Identification of Internal Reference Genes for Gene Expression Normalization between the Two Sexes in Dioecious White Campion

**DOI:** 10.1371/journal.pone.0092893

**Published:** 2014-03-27

**Authors:** Niklaus Zemp, Aria Minder, Alex Widmer

**Affiliations:** 1 ETH Zurich, Institute of Integrative Biology (IBZ), Zürich, Switzerland; 2 ETH Zurich, Genetic Diversity Centre (GDC), Zürich, Switzerland; Naval Research Laboratory, United States of America

## Abstract

Quantitative real time (qRT)-PCR is a precise and efficient method for studying gene expression changes between two states of interest, and is frequently used for validating interesting gene expression patterns in candidate genes initially identified in genome-wide expression analyses, such as RNA-seq experiments. For an adequate normalisation of qRT-PCR data, it is essential to have reference genes available whose expression intensities are constant among the different states of interest. In this study we present and validate a catalogue of traditional and newly identified reference genes that were selected from RNA-seq data from multiple individuals from the dioecious plant *Silene latifolia* with the aim of studying gene expression differences between the two sexes in both reproductive and vegetative tissues. The catalogue contains more than 15 reference genes with both stable expression intensities and a range of expression intensities in flower buds and leaf tissues. These reference genes were used to normalize expression differences between reproductive and vegetative tissues in eight candidate genes with sex-biased expression. Our results suggest a trend towards a reduced sex-bias in sex-linked gene expression in vegetative tissues. In this study, we report on the systematic identification and validation of internal reference genes for adequate normalization of qRT-PCR-based analyses of gene expression differences between the two sexes in *S. latifolia*. We also show how RNA-seq data can be used efficiently to identify suitable reference genes in a wide diversity of species.

## Introduction

RNA-seq, a transcriptome-based next-generation sequencing technique, has revolutionised genome-wide gene expression analysis and has massively increased the number of studied genes and species. In comparison to microarrays for example, RNA-seq has two major strengths [1]. First, the approach is not biased by predefined probes, as is potentially the case in microarray experiments, and is therefore not limited by the availability of microarrays for the study organisms. Second, gene sequence variation can be assessed simultaneously with gene expression variation, thus facilitating the identification of genes with interesting expession patterns, particularly in species with hitherto uncharacterised genomes. Gene expression, however, is an inherently quantitative trait and adequate estimates of gene expression variation may require a level of replication that cannot easily be achieved with large and costly whole-transcriptome analyses. In contrast, quantitative real time (qRT)-PCR is an often used method for studying expression patterns of single genes because of the high sensitivity and specificity [2]. Consequently, qRT-PCR has become the method of choice for the validation of candidate genes identified in RNA-seq experiments with an increased sample of individuals and replicates.

For accurate expression estimation of target genes, expression estimates are normalised relative to internally expressed reference genes. These must be equally expressed among the states of interest for normalization to reduce non-specific variation. Such variation may be the result of different amounts of template RNA or different efficiencies in cDNA synthesis, for example. In addition, suitable reference genes should be expressed at intensities similar to those of the target genes. Typically, two to four references genes are recommended for adequate qRT-PCR data normalization [3], [4]. Traditionally, normalization is done using a single housekeeping gene, such as actin or elongation factor 1-α [2]. However, several studies have revealed that these traditional reference genes are often not as stably expressed as expected, thus leading to misinterpretation of biological data [5], [6]. Consequently, new and more stably expressed reference genes have been successfully identified in a diversity of organisms [7]–[9]. For detailed analyses of gene expression variation between different states (e.g., between different experimental conditions or between different sexes), it is therefore important to systematically screen for appropriate reference genes before embarking on large-scale qRT-PCR analyses.

Reference genes in species with uncharacterized genomes have been identified using microarray data [10], EST databases [11], systematic comparisons of previously used reference genes [12], or a combination of these approaches [13]. With the recent advance in sequencing technology, RNA-seq data can now be directly used to generate *de novo* reference transcriptomes [1] that can be used to identify the most stably expressed genes or contigs on an exome-wide scale. The joint availability of expression and sequence information further facilitates the design of qRT-PCR assays.

Several different algorithms are available for assessing the stability of reference genes using qRT-PCR, such as geNorm [14], Δct [15], NormFinder [16], and BestKeeper [17]. These algorithms help find the most stably expressed reference genes by minimizing the variance across samples. However, the stability rank assigned to different genes by these methods is often different. RefFinder (available at www.leonxie.com/referencegene.php?type=reference) takes into consideration results from all algorithms to arrive at a robust stability estimate and thus further facilitates the identification of suitable reference genes.

Novel reference genes have recently been characterized in several plant species including model [8] and non-model organisms [18], as well as in different tissues [19], at different developmental stages [20], and under different experimental conditions [7]. To date, however, no novel reference genes have been characterized between different sexes. White Campion (*Silene latifolia* Poiret), a dioecious plant with evolutionarily young sex chromosomes, has become a model system for studies of plant sex chromosomes and the evolution of separate sexes [21]. Relatively few gene expression studies have been published for this species. These studies used either Northern blotting [22] or qRT-PCR, typically with a single reference gene, such as GTPase [23] or 18S rRNA [24]. More recently, several studies have used the RNA-seq method to explore gene expression differences between male and female plants [25]–[27]. Consequently, there is a need for good reference genes that allow for an accurate qRT-PCR normalization of gene expression differences between male and female *S. latifolia* plants.

In the present study, we used RNA-seq data from multiple male and female individuals to identify the most stably expressed genes (e.g., contigs of the *de novo* reference transcriptome) in male and female flower buds of *S. latifolia*. The qRT-PCR expression stability of these newly developed reference genes was then compared to traditional reference genes in flower buds and leaf tissues. Finally, we used the best reference genes to estimate the percentages of sex-biased genes in reproductive and vegetative tissues of *S. latifolia* by comparing a set of eight randomly chosen sex-linked genes with sex-biased expression patterns [25].

## Materials and Methods

### Identification of novel reference genes

We used two different RNA-seq datasets derived from flower buds. One included three males and three females of a 10-generation inbred line (U10) [25]. The other dataset contained four females and four males from an intraspecific cross (C1) between an individual from the inbred line (U10) and an outcrossed individual from Leuk (Valais, Switzerland) (unpublished). Paired-end reads were mapped onto the reference transcriptome [25] using BWA [28], allowing for up to five mismatches per read. Of the 141,855 contigs, we removed those that shared similarities with repeated elements and those that never reached ≥10 reads per contig, resulting in 107,002 contigs. FRPKM (fragments per kilobase of exon per million fragments mapped) were calculated and averaged across the two sexes. Then, coefficients of variation (CV  =  standard deviation/mean) between males and females were calculated per contig for the U10 and C1 individuals using a customized script in R [29]. For both datasets, contigs were ranked by increasing CV and the 1% most stably expressed contigs (i.e., those with lowest CV values) between males and females were determined. As candidate reference genes we selected those contigs that were present in both subsets. Sequences of newly identified candidate reference genes have been deposited at DDBJ/EMBL/GenBank under the accession GARX01000000 (Table 1).

**Table 1:** Gene names, primer sequences, amplicon characteristics of the *S. latifolia* candidate reference genes, accession numbers of the protein with the best blast hits in *A. thaliana* and accession numbers of sequences that were used to design the assays are shown.

Gene symbol	Primer sequences (5′-3′)	Amplicon length (bp)	Tm (°C)	Amplification efficiency	Best blast hit	GenBank accession number
SL_REF1	F: TCCTCGGAAGGTTCAAGGGTGTCTT R: TTGGTATCGGTTGGCGGGAGTTTTC	102	59.4	1.89	no hit	GARX01000000 (contig_1076.1)
SL_REF2	F: GCAGTGGTTGTAGTCCGGCATTAGT R: GAGTGTTGCGGTGGAGAGATTGCTT	124	59.3	1.89	no hit	GARX01000000 (contig_12371.1)
SL_BXL4	F: TTTGCTCCTTGCATCGCGGTTTGT R: ACACCCTTTGCGTTATGTGGGAGGT	143	60.4	1.88	Q9FLG1	GARX01000000 (contig_1352.1)
SL_EDL16	F: GGGGCCAATTTCACTTGATGCTGGA R: TAATCCGCCTCGGATACTGGTTGGT	118	59.5	1.85	Q8LBI9	GARX01000000 (contig_13862.1)
SL_METL1	F: TCCGGTGGTTGGGTTCCTCCTAAAA R:GCCGCATGCCAGTGTCAACAAAA	116	59.5	1.77	Q94AI4	GARX01000000 (contig_15898.1)
SL_REF3	F: CGCCAGGCAGAGGTGTTAAACCAGA R: TAGCAGCAGTTACGAGCCCCAACA	145	60.5	1.77	no hit	GARX01000000 (contig_17318.1)
SL_REF4	F: AAAGCGACGATCTTAGGGCGGTTTG R: TCCCCATGTTTGGAGAGGAACTGCT	153	59.7	1.87	no hit	GARX01000000 (contig_18234.1)
SL_REF5	F: TTCCAGGCCCTTAGTGTTAGGGGTT R: AGGGGGAGCTAGCTAAGTGACTTCC	176	59	1.89	no hit	GARX01000000 (contig_18486.1)
SL_PREP1	F: CGCCTCCGCCTTATCTTCGTCATTT R: CTCAACCACCTGCTCGGACACTTTT	178	59.4	1.89	Q9LJL3	GARX01000000 (contig_2002.1)
SL_PIP5K8	F: ACTCCAACGGCGACCCAAAAGAAA R: TAGACGAATCTGCGCTCGTCCTCTT	187	59.5	1.89	Q8RY89	GARX01000000 (contig_2735.1)
SL_COQ3	F: ACGGTCTACGCTTTGCCATCACTTC R: AACACCTGTCACCGTAGCTCCCA	142	59.6	1.86	O49354	GARX01000000 (contig_3547.1)
SL_BL52	F: TGGTTTTGTCCCCACCGAAACGAA R: TATCCCCATCTGGTTGAGCGGTTCT	124	59.2	1.84	O22232	GARX01000000 (contig_38083.1)
SL_RPM1	F: AGAGTGTATGTCTGCCAACTGCCCT R: CGGGGAAAGCGAGAATTTGGAGGTT	183	59.6	1.88	Q39214	GARX01000000 (contig_38634.1)
SL_PDXK	F: TCATCAATGGCGCAACCTCCGATT R: ACCGGGTCAACATCAAAACCAAGCA	152	59.4	1.82	Q8W1X2	GARX01000000 (contig_39927.1)
SL_ASPL1	F: ACGCCGGCTTTGTTGTTCATCAGT R: ATGCAATTCGGGTGGTCTCGTTCAG	108	59.7	1.90	Q9LX20	GARX01000000 (contig_49970.1)
SL_REF6	F: AACTCCCTGTTCTCACCCCATTCCT R: AATCTTCAGTTGCGGAGGGGCTTAC	100	59.1	1.91	no hit	GARX01000000 (contig_56060.1)
SL_BLUS1	F: CCACCACTATGGGATCGCGTGAAA R: TTCCAGGCACTGACACCTCTCTTGT	131	59.3	1.81	O23304	GARX01000000 (contig_57265.1)
SL_STR1	F: ACCCAGTGCAAGAACACAAGCAGT R: ACGGAAGGGCATTAGGAGTGGTCAT	193	59.4	1.84	O64530	GARX01000000 (contig_57652.1)
SL_AKT6	F: TGACTCGGGATCAGCCAGTTCACA R: TTCATCATGCAGCCCGAGGTGAG	122	59.3	1.84	Q8GXE6	GARX01000000 (contig_62745.1)
SL_REF7	F: AGTTGGTACTTGGCGTTTGAGGGAC R: AGGCTCCCTAGGAAACAGTCGGAAA	185	59.1	1.83	no hit	GARX01000000 (contig_64521.1)
SL_REF8	F: GCTTGTGGAAGCAGGGGATCTTTGT R: AGGGCCAGTTTCCGCATGATTTGT	174	59.4	1.91	no hit	GARX01000000 (contig_25240.1)
**SL_TUB**	F: CTGGGAAATACGCAGGTGAT R: ATTCCCAGCACCAGATTGAC	200	59.9	1.90	NP_564101	GH293053
**SL_ACTIN**	F: CTGGTTTCGCTGGAGATGAT R: GGGTTCAATGGTGCTTCTGT	280	60.1	1.89	NP_187818	GH293703
**SL_UBCE**	F: TTCATTGCTTGCCACTTCTG R: CAAATGCGAGCTGAAAAACA	201	60	1.86	NP_568476	GH294915
**SL_EF1**	F: GGCCACTTTCTGCTCTGGTA R: GGTCTTCACGGACACTGGTT	182	59.8	1.84	AAL91176	GH294786
**SL_GAPDH**	F: GCGAGACTGGAGCTGATTTC R: ACAACTGGCATTGGACACAA	194	60.1	1.87	NP_172801	GH291995
**SL_UBQ**	F: AATTTTCGCCTTCCTCATCC R: GCTTGCCAGCGAAAATAAGT	396	60	1.80	NP_567286	GH293574

Gene names of traditional reference genes are written in bold.

Candidate reference gene sequences were annotated using the *Arabidopsis thaliana* entries of the Uniprot database [30] (Table 1). qRT-PCR primer assays were designed using Primer3 [31] and the parameters recommended by [32]. The assays were designed as follows: length of primers 24–25 base pairs (bp) and of amplicons 100–200 bp; GC-content between 50 and 60%; annealing temperature 60±1°C; temperature difference between the two primers <0.5°C; hairpin formation parameter ≤6 and primer dimer and pair dimer parameter ≤4. Additionally, we took the mapped RNA-seq reads across all individuals into account to identify only conserved primer binding regions and amplicons with equally distributed RNA-seq reads. Primer assays for the traditional reference genes were designed from EST sequences [33] that were blasted against characterised protein sequences [30]. The following traditional reference genes were identified in our RNA-seq reference sequences by blasting them against the Uniprot database [30]: *actin 11* (SL_ACTIN), *putative ubiquitin-conjugating enzyme E2 21* (SL_UBCE), *tubulin beta-5 chain* (SL_TUB*), eukaryotic translation initiation factor* (SL_EF1), *ubiquitin 11* (SL_UBQ), and *glyceraldehyde-3-phosphate dehydrogenase* (SL_GAPDH).

### qRT-PCR validation using reproductive and vegetative tissue

Four flower buds (5–6 mm) without the calyx and one disc (1 cm diameter) of a young, fully developed rosette leaf were collected from three males and three females of an 11-generation inbred line (U11); similarly, four males and four females were collected from the C1 cross. All samples were snap-frozen in liquid nitrogen directly after sampling. Total RNA was extracted from both tissue types separately using the RNeasy Plant extraction kit (Qiagen, USA) with a BeadRuptor (Omni International, USA). RNA was treated in tube (see manufactory's instruction) with a RNase-Free DNase Set (Qiagen, USA) and purified over a second column because we observed that on-column digestions were not sufficient to eliminate genomic DNA contaminations. RNA was checked for genomic DNA contamination by amplifying an intron-spanning PCR product whose fragment size was checked on agarose gels. The integrity of the RNA was assessed on an Agilent 2100 Bioanalyzer Plant RNA chip (RIN ≥7.5 for leaves and ≥9 for flower buds) and RNA concentrations were determined using a Qubit Fluorometer (Life Technologies, USA). 0.5 μg total RNA was then reverse transcribed to cDNA according to the standard protocol using the QuantiTect Reverse Transcription Kit (Qiagen, USA) including the genomic DNA Wipeout buffer. qRT-PCR reactions were performed in triplicates on a 7500 Fast Real Time PCR system (Life Technologies, USA) using SYBR FAST Universal 2X qPCR mix with low ROX concentration (Kapa Biosystems, USA) in a total volume of 11 μl. To assess the quality of our primer assays, we tested for dimer formation and the absence of amplification products in NTCs (no template controls), and Sanger sequenced (3730xl DNA Analyzer, ABI) all amplified products to confirm amplification of target transcripts. Transcripts were considered undetermined if their cycle threshold (C_t_) values exceeded 35. In cases of signals in NTC, the difference between the C_t_ of NTC and that of the template had to be ≥8 C_t_ values. Primer assays that produced ≥10% missing data were excluded. The remaining gene assays were ranked according to their expression stability between males and females in flower buds and leaves (25 and 26 candidate reference genes in flower buds and leaves, respectively). We used the web tool RefFinder to assign robust ranks based on the results from the four widely used stability estimators geNorm, Δct, NormFinder, and BestKeeper. We used all data, including the technical replica, to calculate stabilities. Results from one leaf sample were consistently identified as outliers and were therefore removed. Consequently, we randomly removed one leaf sample of the other sex to maintain a balanced design. qRT-PCR efficiencies were calculated across all samples and tissues using LinReg [34].

### Sex-biased gene expression in reproductive and vegetative tissues

We used the same cDNAs as for the reference genes and eight randomly selected sex-linked genes that were previously found to have sex-biased gene expression patterns (five genes with male-biased and three with female-biased expression), based on RNA-seq data [25]. Primer assays were designed and qRT-PCRs were performed as described above. We normalized the expressed intensities separately for the two tissues using SL_GAPDH, SL_PIP5K8 and SL_REF2 as reference genes for analyses of flower buds and SL_PDXK, SL_REF6 and SL_UBCE for leaves. According to RefFinder, these were the most stable reference genes among those with similar expression levels as the genes of interest. Normalization was performed with individual qRT-PCR efficiencies using QBasePlus [35]. We then validated the stabilities of these reference gene combinations using geNorm estimates and found that both M and the pairwise variation were smaller than 0.5 and 0.15, respectively, indicating stable expression [36]. Results remained stable when we normalized data over all tissues and sexes (data not shown). Technical variations among qRT-PCR triplicates were negligible (mean standard deviation <0.3 C_t_) and were therefore not used further in the statistical analysis. Outliers were removed (<25th or >75th percentile) and fold changes and mean standard errors over the 5-7 biological replicates per sex were calculated using R. We considered contigs to be significantly differentially expressed between males and females if fold changes were ≥1.5 and Wilcoxon tests remained significant after performing Benjamini-Hochberg corrections for multiple testing (p≤5%) [37].

## Results

### Identification of candidate reference genes based on RNA-seq data

Only a small proportion of contigs from across the transcriptome are suitable for use as reference genes because of the high coefficients of variation (CV) observed for most contigs (Figure S1). We found 24 contigs that were consistently amongst the 1% most stably expressed contigs between the two sexes and the two RNA-seq datasets, and these contigs showed a broad expression range (0.3-92.3 FRPKM) in *S. latifolia* flower buds., We could not design primer assays for two of these contigs, and one of the 22 validated candidate reference genes was excluded because it did not pass our quality control (see Material and Methods) (Table 1).

### Candidate reference genes in reproductive and vegetative tissues

**Figure 1:**
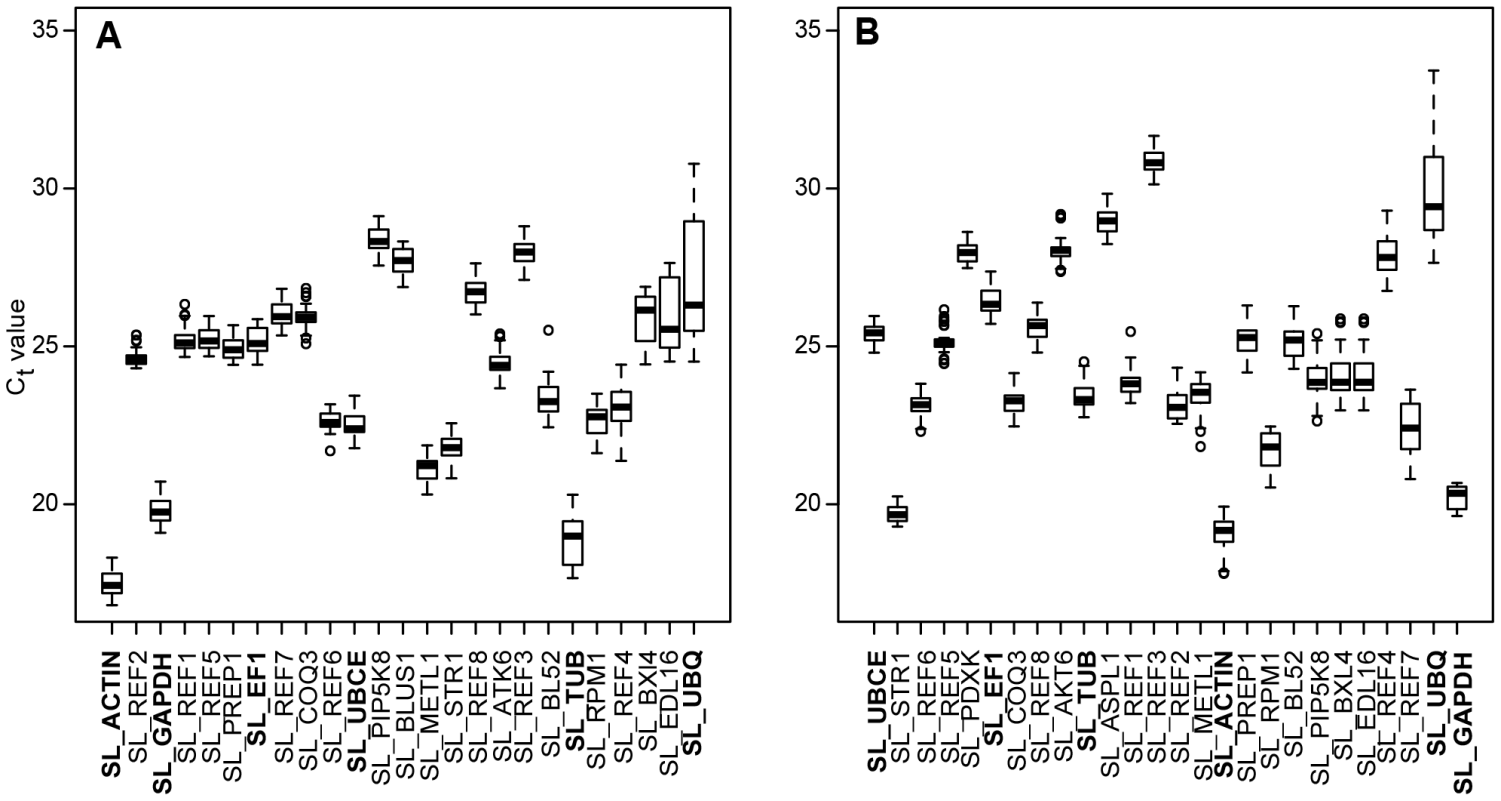
Expression (C_t_) values of candidate reference genes ordered by decreasing stability in reproductive and vegetative tissues. Boxplots represent C_t_ values over all tested samples in (A) flower buds (25 genes) and (B) leaves (26 genes) for candidate reference genes that are arranged by decreasing (left to right) stability rank as inferred from RefFinder. Traditional reference genes are indicated in bold.

**Figure 2:**
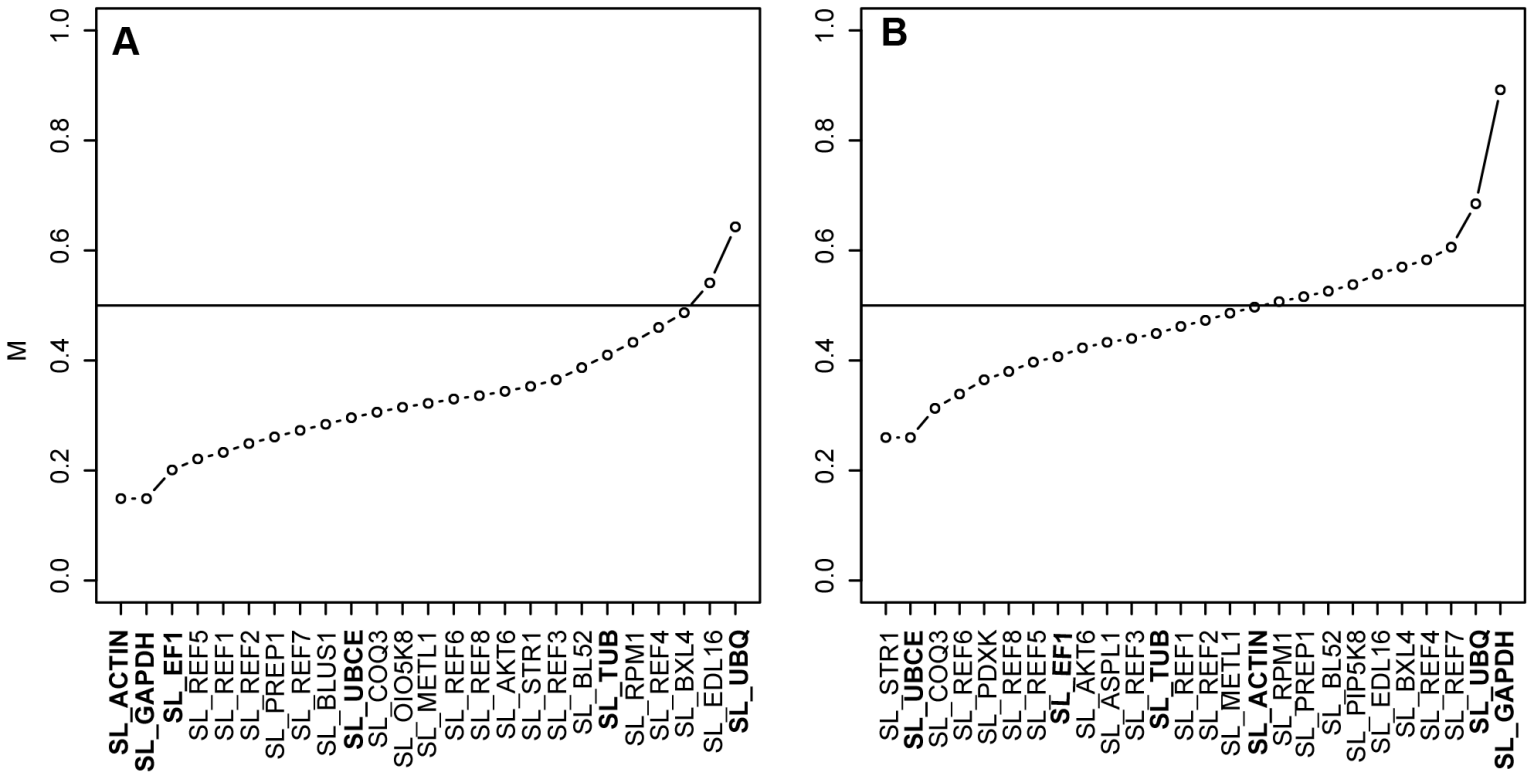
Gene expression stabilities of candidate reference genes. Average expression stabilities (M) of the candidate reference genes in (A) flower buds (25 genes) and (B) leaves (26 genes) as estimated by geNorm. Small M values indicate high stability. M values for most genes were below 0.5, indicating high expression stability [36]. Traditional reference genes are indicated in bold.

The five most stably expressed genes between the sexes, as determined by RefFinder, were SL_ACTIN, SL_GAPDH, SL_REF1 SL_REF2, and SL_REF5 in flower buds (Fig. 1A), and SL_PDXK, SL_STR1, SL_UBCE, SL_REF5 and SL_REF6 in leaves (Fig. 1B). There were slight differences in the stability ranks of the genes across the four ranking methods, such as the M values of geNorm (Fig. 2) and the stability ranks of RefFinder (Fig. 1).

Based on the RNA-seq data from flower buds, most traditional reference genes were found among the 5% most stably expressed contigs. In one of the datasets, SL_EF1 was found to be among the 1% most stably expressed contigs (Table S1). qRT-PCR data revealed that in both tissues some of the traditional reference genes were among the five most stably expressed contigs: SL_ACTIN and SL_GAPDH in flower buds and SL_UBCE in leaves of *S. latifolia*. However, SL_ACTIN and SL_GAPDH had particularly low C_t_ values as a consequence of their strong expression in the studied tissues (Fig. 1A).

qRT-PCR data of the newly identified candidate reference genes showed similar or slightly reduced stabilities when compared to traditional reference genes, but their expression intensity ranges were larger across the different genes in both tissues (Fig. 1). Mean differences among the M values of the candidate reference genes were small: ΔM = 0.021±0.004 and ΔM = 0.025±0.008 for flower buds and leaves, respectively (Fig. 2). Out of the 29 reference genes tested, four were excluded because they did not pass our quality control and two because they lacked sufficiently stable expression, leaving us with 23 reference genes (18 novel and five traditional ones). Depending on the expression intensity of the genes of interest, these 23 reference genes can be used for accurate normalization of expression estimates in flower buds (Fig. 1A).

In leaves, three assays did not pass quality controls and stability thresholds, leaving 16 (eleven novel and five traditional ones) reference genes (Fig. 1B). The successful identification of reference genes for analysis in vegetative tissues is particularly interesting because candidate reference genes were originally identified from RNA-seq data derived from flower buds. Across both tissue types, the five most stably expressed candidate reference genes (SL_PREP1, SL_REF2, SL_REF5, SL_REF6, and SL_REF8; see Figure S2A) were all newly identified reference genes (Figure S2).

### Sex-biased expression in vegetative and reproductive tissues

**Figure 3:**
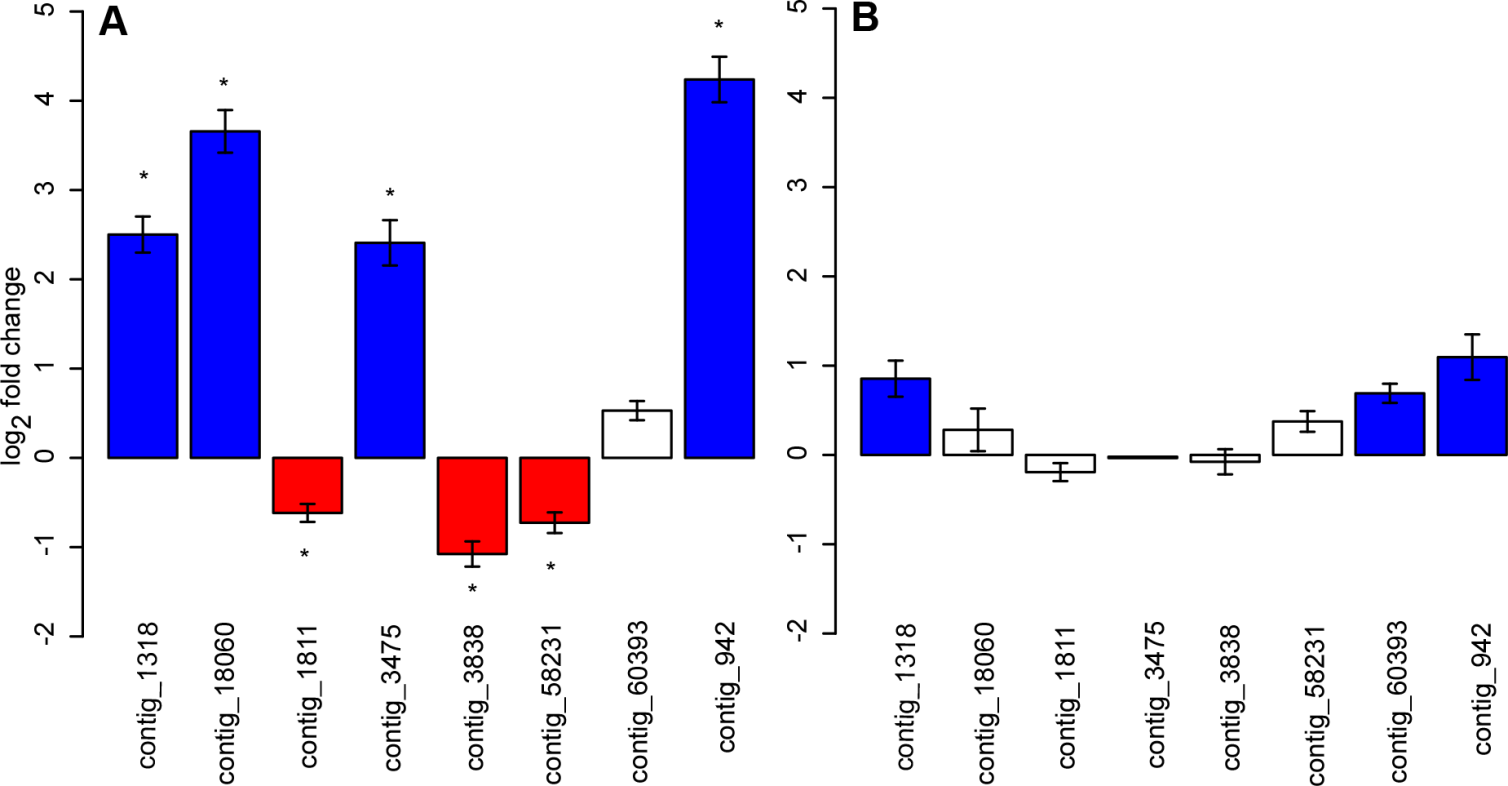
Sex-biased expression of eight target genes in vegetative and reproductive tissues. Transcript levels are shown as log_2_ fold changes for eight sex-linked genes in (A) flower buds and (B) rosette leaves. Red and blue colours indicate female- and male-biased expression (fold change ≥1.5), respectively. Asterisks indicate significant expression differences between males and females based on Wilcoxon tests after correction for multiple testing. Error bars indicate mean standard errors calculated from five to seven biological replicates normalized over the three selected reference genes. No expression was observed for contig_3475 in leaf tissues.

Seven of the eight contigs that were originally inferred to have sex-biased expression based on RNA-seq data [25] were also found to have significantly sex-biased expression in flower buds based on qRT-PCR results. One of the contigs with male-biased expression showed the expected direction of sex-bias in the qRT-PCR analysis, but the difference between the sexes was not statistically significant. As previously determined from RNA-seq data, four of the contigs had significantly male-biased expression, and three were female-biased (Fig. 3A). In leaves, seven of the eight genes were expressed but none was significantly sex-biased, although three contigs showed a trend toward male-biased expression (Fig. 3B).

## Discussion

RNA-seq data allow the analysis of thousands of genes in one single experiment and make it possible to screen the transcriptome for candidate genes showing expression patterns of interest. qRT-PCR is an effective, robust and inexpensive method for the subsequent validation of such RNA-seq experiments and for detailed studies of identified candidate genes. Medium and high throughput qRT-PCR experiments typically allow inclusion of more biological and technical replica compared to RNA-seq based transcriptome analyses. For an appropriate normalization of the qRT-PCR data it is, however, essential to have two to four suitable reference genes available in order to reduce non-specific expression variation that can otherwise lead to incorrect biological conclusions [3], [4]. Thus, it is important to identify appropriate reference genes for qRT-PCR studies before embarking on large-scale expression analysis.

The identification of novel reference genes in species with hitherto uncharacterized genomes can be difficult because nucleotide sequences for primer design and the transcriptional response of available genes to the experimental conditions may be unknown. In contrast, the increasing number of available transcriptome data from RNA-seq experiments, especially in many non-model organisms, now allows *de novo* assembly of reference transcriptomes that can be searched for the most stably expressed contigs. In this study, we observed that only a small proportion of contigs could be used as candidate reference genes because expression of the great majority of genes varied substantially between biological replica and the two sexes. It is therefore important to evaluate a large number of expressed genes for their suitability as reference genes. The approach we used here to identify reference genes was straightforward. We used two different RNA-seq datasets from multiple individuals to reduce technical variation, and population effects to identify candidate reference genes that span a broad expression range. These genes (Table 1) are mainly involved in cellular processes and have housekeeping functions. A recent study further revealed that expression stabilities based on RNA-seq and qRT-PCR experiments are highly correlated [38]. Thus, RNA-seq data can be successfully used to identify reference genes in organisms with weakly characterised genomes.

Assay design for reference genes can be difficult and time-consuming. Traditional reference genes are often members of large protein families, which make the design of specific primers difficult, especially when gene families have not been previously characterized [39]. Reference gene assays based on ESTs or customized microarray data may therefore not fulfil the criteria for good qRT-PCR assays. The MIQE guidelines [3], [4] and the Eleven Golden Rules of Quantitative RT-PCR [32] emphasize the importance of carefully checking all qRT-PCR assays: (i) perform melting curve analyses to detect unspecific products or dimer formation, (ii) verify target transcripts using Sanger sequencing, (iii) check for signals in NTCs and (iv) achieve reasonable qRT-PCR efficiencies. In this study, just one out of 22 newly developed assays did not pass quality control. The availability of nucleotide sequence and expression information from RNA-seq experiments strongly facilitated the design of qRT-PCR assays. Interestingly, more than half of the candidate reference genes identified based on RNA-seq data from reproductive tissue can also be used for qRT-PCR normalization in vegetative tissues. The reference genes identified in this study may therefore also serve as a starting point for the identification of reference genes suitable for further gene expression comparisons, such as between different experimental conditions or tissue types.

Our results from *S. latifolia* males and females revealed that the newly identified reference genes were more stably expressed than traditional reference genes across both tissue types. When flower buds and leaves were considered separately, some of the traditional reference genes were equally or even more stably expressed than the newly identified reference genes. Similar observations were made in other studies in which traditional reference genes were also found to be more stably expressed than newly identified ones [10], [40], [41]. However, other studies have found the opposite [7]–[9]. This inconsistency between species and experimental conditions suggests that it is important to carefully validate and compare both traditional and newly developed reference genes. In addition to stable expression, reference genes ought to have expression intensities similar to those of the target genes [42]. We observed that most traditional reference genes were strongly expressed, whereas candidate genes showed a much broader expression range. Our catalogue of more than 15 newly developed and traditional reference genes allowed us to choose a suitable combination of reference genes with respect to both expression stability and intensity. For our comparison of the intensity of sex-biased expression in flower buds and leaves, three reference genes were sufficient for adequate normalization of qRT-PCR data. The stability of the selected reference genes, however, remains to be validated prior to expression normalization in each case study.

As recommended in the MIQE guidelines [3], [4], it is important to use high quality RNA for expression analyses. Partly degraded RNA can have a strong influence on expression estimates. To assess the effect of RNA quality on the stability of gene expression estimates, we used multiple RNA samples from flower buds that were extracted as described above (see Materials and Methods) one and two years earlier than the samples used in this study. These earlier extractions were maintained at a constant −80°C during this period. In both samples, the ribosomal subunits were intact and RNA quality, as assessed with the Bioanalyzer RNA chip, was found to be good (RIN ≥9). cDNA synthesis followed the same protocol as above. However, we found clear evidence for reduced expression stability in the older RNA samples (Figure S3), suggesting that ribosomal RNAs in RNA extractions from *S. latifolia* are more stable than long coding RNAs. Hence, reference genes should ideally be validated with freshly extracted, high quality RNA. These reference genes can further be used to test sample RNA quality after storage and prior to expression analysis.

*S. latifolia* is a dioecious plant with a chromosomal sex determination system and heterogametic males. The heteromorphic sex chromosomes evolved less than 10 million years ago [43] and are younger than most animal sex chromosome systems. Nevertheless, *S. latifolia* sex chromosomes show several characteristics of these older animal systems, including reduced expression of the alleles on the Y chromosomes [26], [27], [44], and evidence for dosage compensation [25]. *S. latifolia* is therefore suitable for studying expression phenomena on young sex chromosomes. To date, targeted expression studies in this species used Northern blotting [45]–[48] or qRT-PCR experiments in which expression of target genes was normalized with a single, non-validated reference gene [24], [49]. The reference genes presented in this study allow for the adequate normalization of qRT-PCR data and will hopefully contribute to a better understanding of gene expression differences between *S. latifolia* males and females in both reproductive and vegetative tissues.

It has been postulated that sexual dimorphism is mainly a result of expression differences between males and females [50]. In this study, we tested eight genes that were previously inferred to have sex-biased expression based on RNA-seq data. Our qRT-PCR experiments confirmed these results. Expression differences between the sexes were observed for all eight genes, and expression differences between males and females were statistically significant for seven of these genes in reproductive tissues (flower buds). In contrast, none of these genes had a significant sex-bias in expression in vegetative tissues (rosette leaves). These results point towards a reduced sex-bias in expression of sex-linked genes in vegetative tissues (rosette leaves) of *S. latifolia*. This reduction or shift in sex-bias in somatic tissues has also been found in animal systems [51]–[53]. Interestingly, most of the investigated genes in *S. latifolia* were also expressed in vegetative tissues, and three of the seven expressed genes showed a trend toward male-biased expression. In vegetative *S. latifolia* tissues, few genes have been studied to date, presumably genes that are not located on the sex chromosomes, suggesting that expression differences between males and females also exist in leaves [22]. Indeed, there are sexually dimorphic traits in *S. latifolia* leaves [54]. However, for determining the proportion of genes with sex-biased expression on a genome-wide level in vegetative tissues, one has to massively increase the number of studied genes by using, for example, an RNA-seq approach.

In summary, we present in this study a validated catalogue of newly identified and traditional reference genes for gene expression analyses in *S. latifolia*. These are suitable for normalizing gene expression estimates from qRT-PCR studies of vegetative and reproductive tissues in males and females of *S. latifolia*. Furthermore, we used some of these newly identified reference genes to confirm sex-biased expression of eight target genes in flower buds and found evidence for reduced sex-bias expression in vegetative tissues.

## Supporting Information

Figure S1 Genome-wide expression stability estimates of genes in flower buds of male and female *S. latifolia* based on RNA-seq data. Contigs are ordered by increasing CV [0-1] between male and female flower buds based on RNA-seq data for (A) individuals from an inbred line (U10) and (B) individuals from an intraspecific cross. Low CV values indicate high expression stabilities between the sexes. The 1% most stably expressed contigs are highlighted in green (bottom left of A and B).
(TIF)

Figure S2 Expression values (C_t_) and stabilities of candidate reference genes ordered by decreasing stability across both sexes and tissue types. (A) Boxplots of **C_t_** values across all tested samples and tissues for candidate reference genes arranged by decreasing (left to right) stability rank as inferred from RefFinder. (B) Average expression stability (M) estimates for all candidate reference genes across all samples and both tissue types. Traditional reference genes are indicated in bold.
(TIF)

Figure S3 Gene expression stabilities of candidate reference genes depend on RNA quality. Average expression stabilities of candidate reference genes in cDNA from flower buds after long-term storage (two years) at −80°C (A) and short-term storage (one year) at −80°C (B).
(TIF)

Table S1 Traditional reference genes found among the 10%, 5% and 1% most stably expressed contigs between male and female flower buds based on RNA-seq data.
(XLSX)
