# Supplementary figures and images for: Identification of Internal Reference Genes for Gene Expression Normalization between the Two Sexes in Dioecious White Campion
Source: PLoS One. 2014 Mar 27;9(3):e92893. doi: 10.1371/journal.pone.0092893 (PMC3968030; doi:10.1371/journal.pone.0092893)

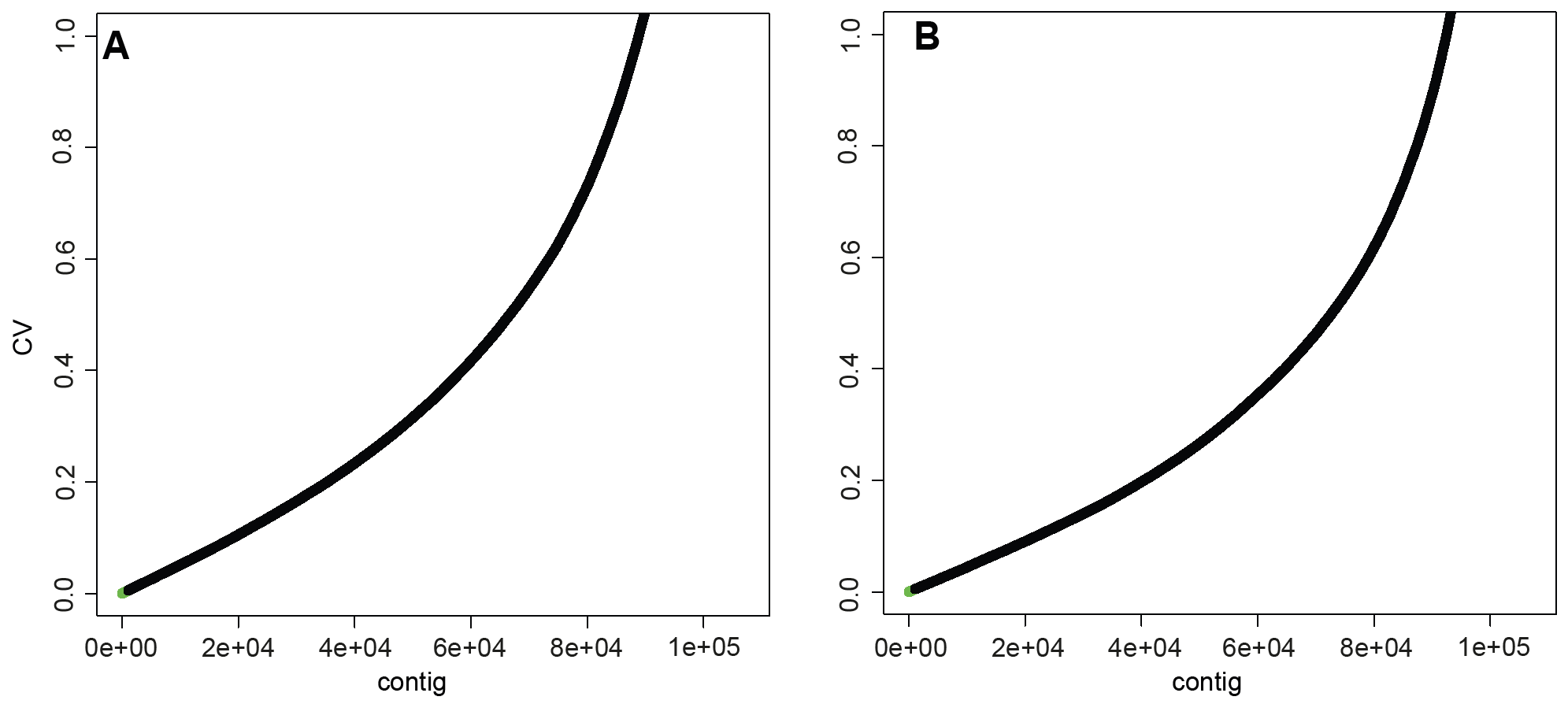

Supplement: Figure S1 — Genome-wide expression stability estimates of genes in flower buds of male and female S. latifolia based on RNA-seq data. Contigs are ordered by increasing CV [0-1] between male and female flower buds based on RNA-seq data for (A) individuals from an inbred line (U10) and (B) individuals from an intraspecific cross. Low CV values indicate high expression stabilities between the sexes. The 1% most stably expressed contigs are highlighted in green (bottom left of A and B). (TIF) [file pone.0092893.s001.tif]

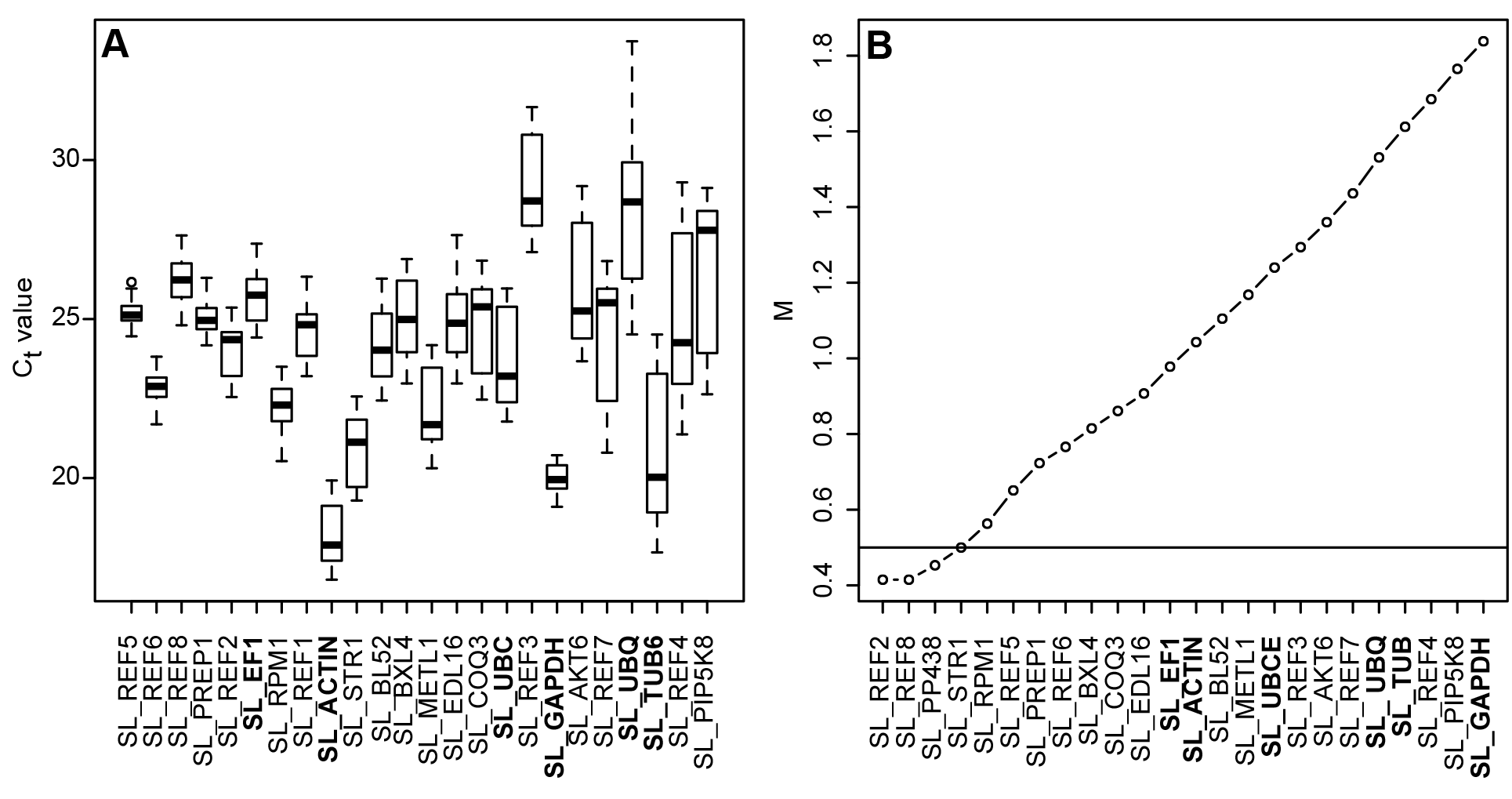

Supplement: Figure S2 — Expression values (Ct) and stabilities of candidate reference genes ordered by decreasing stability across both sexes and tissue types. (A) Boxplots of Ct values across all tested samples and tissues for candidate reference genes arranged by decreasing (left to right) stability rank as inferred from RefFinder. (B) Average expression stability (M) estimates for all candidate reference genes across all samples and both tissue types. Traditional reference genes are indicated in bold. (TIF) [file pone.0092893.s002.tif]

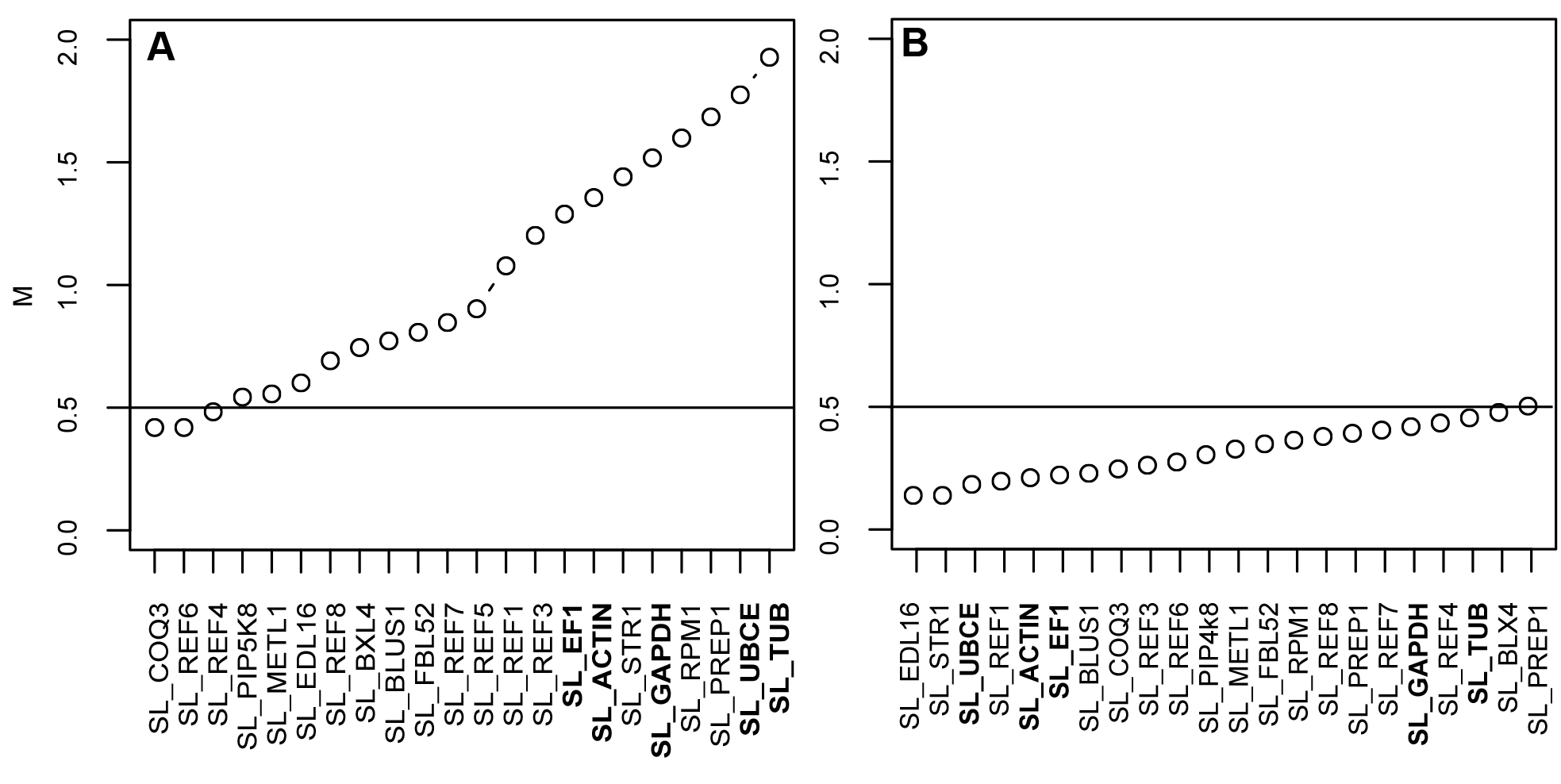

Supplement: Figure S3 — Gene expression stabilities of candidate reference genes depend on RNA quality. Average expression stabilities of candidate reference genes in cDNA from flower buds after long-term storage (two years) at −80°C (A) and short-term storage (one year) at −80°C (B). (TIF) [file pone.0092893.s003.tif]
